# A Pilot Study to Establish the Leadership Development Needs of Community Pharmacist Leads in Lambeth, South East London

**DOI:** 10.3390/pharmacy11040114

**Published:** 2023-07-06

**Authors:** Mohammed Patel, Finlay Royle, Ricarda Micallef

**Affiliations:** 1 Department of Pharmacy, Penrhyn Road Campus, Kingston University London, London KT1 2EE, UK; 2 NHS South East London Integrated Care Board, London SE1 2TZ, UK

**Keywords:** pharmacist, primary care, community pharmacy, leadership, communication, primary care networks, transformation, quality improvement, training, development

## Abstract

Primary care networks (PCNs) are geographical networks consisting of 30,000 to 50,000 patients and groups of general practices working in a multidisciplinary team, including community pharmacists. Community pharmacy (CP) neighbourhood leads act as a conduit between pharmacy contractors and general practitioners (GPs) in these networks, sharing information and providing a voice for the community pharmacy locally. The Lambeth medicines team (NHS South East London Integrated Care Board) recognised the need to continue funding these leadership roles to address barriers to relationship-building between community pharmacies and general practices, the consistency of service delivery and effective communication. The aim of this study was to understand the current experience of CP neighbourhood leads to inform their further development. All eight CP neighbourhood leads individually completed a semi-structured interview over Microsoft Teams, which was then reviewed using content analysis. Ethical approval was received. Leads reported the use of common communication methods such as emails, text messaging applications and telephone calls to engage GPs and pharmacies in their neighbourhoods. Barriers to undertaking their roles included time constraints, delays in responses, high workloads and competing pressures. Other factors impacting their effectiveness and ability to undertake their roles included the scheduling of meetings outside of working hours, finding time during busy working days and organising locum cover on an ad hoc basis. The leads also reported they spent more time focussed on building relationships with their peers and less time focussed on general practice colleagues. Support for CP neighbourhood leads could include ensuring that funded time is protected; communication and technology training; and the provision of more structural support for communication with GPs. The findings of this study can be used to inform future work.

## 1. Introduction

The pressures on health services in advanced economies arising from the global pandemic are immense [1]. The combined effects of significant waiting lists for routine care, workforce shortages, medicines shortages, financial constraints during economic uncertainty, health inequalities and growing demand from populations that are getting older and living with multiple preventable long-term conditions are causing significant challenges [2,3].

In England, the National Health Service (NHS) has set out a number of strategic approaches to transform health services to meet these challenges, aligned with structural changes as outlined in the Health and Care Act 2022 [4]. By delivering on the ambitions detailed in the Fuller stocktake [5], the NHS England primary care recovery plan [6] and the NHS Long-Term Plan [7], Integrated Care Systems (ICS) promise to deliver greater patient-centred care; improved patient outcomes; reduced health inequalities; improved productivity and value for money; and wider economic benefits [8].

Building on their critical role during the COVID-19 pandemic, community pharmacies will play a key role in meeting that ambition. Increasing the shift from a traditional, transactional medicine supply function to a clinically focussed, outcome-orientated service will maximise the opportunities for pharmacists working in community pharmacy settings to utilise their clinical expertise [9].

The potential impact of community pharmacies in managing chronic conditions is significant [7,10,11]. Community pharmacies, located in the heart of the communities they serve, are well placed to deliver clinical services to populations with the greatest need. Ninety-eight per cent of the most deprived neighbourhoods can reach a pharmacy within a 20 min walk of their homes [12]. With a 10-year gap in life expectancy between the lowest and highest Indices of Multiple Deprivation deciles [7], there is an urgent need from a clinical, social and economic perspective to reduce health inequalities and improve outcomes for people at the greatest risk. By increasing capacity, maximising the skills of pharmacists and their teams and providing flexible and accessible premises, pharmacies will play an important role in finding, diagnosing and treating people who are at risk of poor health outcomes arising from high blood pressure, smoking, obesity and other modifiable risk factors.

In 2019, the NHS Community Pharmacy Contractual Framework (CPCF) for England was agreed upon between the Department of Health and Social Care (DHSC), NHS England and the Pharmaceutical Services Negotiating Committee (PSNC), now called Community Pharmacy England, as of June 2023 [13]. The framework outlines the transformative role that community pharmacies will play in meeting the ambition of the Long-Term Plan [7]. The framework recognises the role community pharmacies can play in the prevention, identification and management of long-term conditions such as hypertension, chronic respiratory disease and diabetes with the investment of GBP 13 billion over 5 years, from 2019 to 2024 [7,10,13].

With community pharmacies delivering more clinical services, there is a growing need for joint working with general practices. To provide general practices with infrastructure for integrated care and the delivery of the Long-Term Plan, primary care networks (PCNs) were established in 2019 [7]. These are networks typically covering a geographical region consisting of 30,000 to 50,000 patients and are formed by groups of general practices working in a multidisciplinary team alongside community pharmacies, mental health services, social care services and hospital services. They employ a range of healthcare professionals, for example, general practitioners (GPs), pharmacists, nurses, paramedics, social workers and mental health practitioners. Their purpose is to deliver primary care in partnership with other healthcare providers; social care services; and the voluntary and community sectors [14].

There are around 1250 PCNs in England, and they operate within each of the 42 Integrated Care Systems (ICS) across the country [8]. Since July 2022, Integrated Care Boards (ICB) have replaced the responsibilities of Clinical Commissioning Groups (CCGs), and they hold around 80% of the NHS budget at a local level. This includes additional responsibilities for pharmacy; optometry; dentistry contract management; and, in the future, specialised commissioning [4].

To provide implementation support for community pharmacy integration with general practices and PCNs, NHS England has funded a community pharmacy clinical lead in each ICB, and their role is to help each ICS maximise the delivery of the CPCF, including services such as blood pressure checks and the Community Pharmacy Consultation Service (CPCS), increasing healthcare capacity through the increased utilisation of community pharmacies.

In Lambeth, there is recognition of the opportunities that community pharmacy integration presents. Lambeth is a culturally and ethnically diverse borough in South East London with an estimated 318,000 people speaking over 150 different languages, with 40% from black and multi-ethnic backgrounds [15,16]. The Lesbian, Gay, Bisexual, Transexual Plus (LGBT+) community makes up a significant proportion of the population, with a reported 11% of the population identifying as LGB, against 4% on average across England [16]. A significant proportion of the population is younger, with 61% aged under 40 years, although the rate of growth in the over 65 s is significant, with an increase of 20.1% since 2011 [16,17]. The local authority estimates an increase of 30.4% in the over-60 population by 2032, with an overall growth rate of 5% [15,16]. In England, the Indices of Multiple Deprivation (IMD) provide a measure of relative deprivation at a local-area level for small areas in England and shows that Lambeth is the 11th most deprived borough in London [18,19].

**Figure 1:**
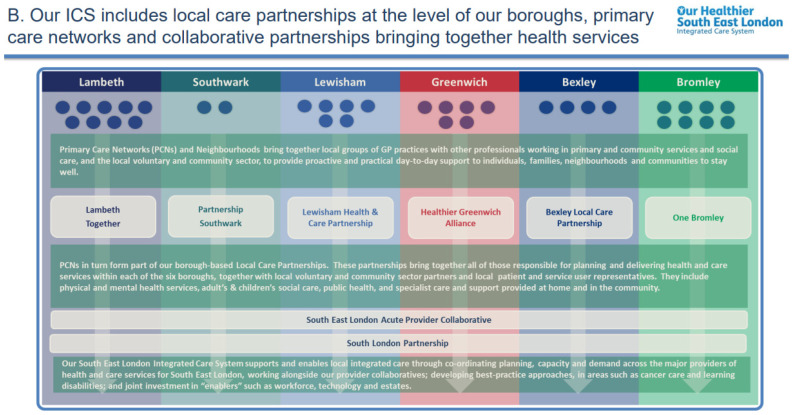
Summary of Integrated Care System structure in South East London [22].

Social deprivation and ethnicity have a significant influence on multimorbidity, and local people in Lambeth suffer from a greater burden of health inequalities as a result. The black African-Caribbean population living in the most socially deprived neighbourhoods develop multimorbidity on average 10–15 years earlier than other populations, and the LGBT+ community has higher rates of both mental and physical ill health and long-term conditions [20,21]. Community pharmacies play an increasingly important role in diagnosing, treating and optimising long-term conditions. Therefore, integrating community pharmacy services is a key priority within Lambeth and South East London. Building on the concept originally identified in CPCF 2019, it was recognised that community pharmacy leadership at the neighbourhood level would be key to embedding an approach that improves both the quality and consistency of pharmacy services and strengthening relationships between general practices and community pharmacies. Lambeth has, therefore, invested in funding community pharmacy (CP) neighbourhood leads, which cover an area approximately equivalent to primary care networks. The CP neighbourhood lead works as a pharmacist in their pharmacy but takes on additional responsibilities as a clinical leader at a local level. Figure 1 shows the health system in South East London.

Supported by the ICB medicines optimisation team, the Local Pharmaceutical Committee and PCNs (through local contracts), these CP neighbourhood leads work with and support community pharmacies in their locality to not only increase the delivery of national and local services but also to improve the quality and consistency of care that they deliver. Funded for four hours per month, they have a role in facilitating an understanding of community pharmacy services and addressing any local challenges through regular communication with the corresponding PCN clinical director. Leads may also identify additional needs to meet the demands of the local population [23]. Thus, the specific roles of a lead can vary depending on the neighbourhood in which they practice; the service goals they have; and the requirements of the local population they serve.

As clinical leaders, CP neighbourhood leads have an important role in communication. In healthcare systems, communication can take place in a complex environment where conditions may not always be favourable, yet the continuous exchange of information must prevail between health and care professionals to meet the demands of patient care [24]. Given the nature of the services pharmacies now deliver, communication is pivotal to delivering effective, joined-up, quality care for local people.

The aim of this study was to evaluate the experience of CP neighbourhood leads as they started in their roles; identify barriers to communication and areas of good practice; understand their learning and development needs; and make recommendations for the further development of the programme.

## 2. Materials and Methods

### 2.1. Study Design 

Qualitative data from CP neighbourhood leads were received using semi-structured, one-on-one interviews. This approach was taken to gain the perceptions of individual practices. An interview proforma consisting of nine questions was designed. It aimed to understand current methods and the content of communication; the barriers and enablers of current communication; establish stakeholders; and establish ongoing support requirements.

The interview schedule received face validation for content from ICB medicines optimisation leads who were not included in the interviews. A copy of the interview schedule can be found in Appendix A. To ensure study integrity in the design and analysis, the COREQ checklist was used. This can be found in Appendix B.

### 2.2. Participants: Sampling and Recruitment

Human participants (n = 8) were involved in data collection through semi-structured interviews.

Potential participants were identified and contacted through the ICB leads of Lambeth, with pharmacy email addresses and contact numbers being provided to the researcher. The researcher had no prior relationship with the CP neighbourhood leads. The CP neighbourhood leads were made aware of the study on two development days and were invited to take part. The study was based on 8 out of 9 neighbourhoods in the London Borough of Lambeth, including Stockwellbeing, AT Medics Streatham, Croxted, Brixton and Clapham Park, Fiveways, HBD, Streatham and North Lambeth. The remaining PCN, Clapham, was excluded from the research as the position of CP neighbourhood lead in this locality was vacant at the time this study was undertaken.

### 2.3. Data Collection

One-on-one interviews were conducted with each of the CP neighbourhood leads in Lambeth from February and March 2023. The interviews were held over Microsoft Teams and telephone and were arranged according to the preference and availability of the pharmacist. Individuals who agreed to participate in an interview were emailed an information sheet outlining the study aims and objectives; the background of the researchers; the right to withdraw from the study; and a consent form, which they were asked to read, sign and return prior to the agreed interview time. Although written consent to record the interviews was provided by each participant via email, additional verbal consent was requested at the start of each interview to ensure participants agreed to be audio-recorded. Interviews lasted between 12 and 30 min. All interviews were voice-recorded and transcribed verbatim prior to deletion. No other notes were made during the interviews. The interviews took place between February 2017 and October 2018. All those who initially agreed to be interviewed completed an interview so that all views and experiences were included. One male member of the research team (MP), who was completing his Master of Pharmacy thesis at the time of the study, completed all the interviews and completed the transcriptions after appropriate training sessions. No other individuals besides the researcher and participant were present during the interviews.

Of the eight interviews conducted, three interviews were conducted online via Microsoft Teams, and to facilitate participation in the interviews, five interviews took place over the telephone. All interview recordings and data were stored on a password-protected system and immediately deleted after transcription using Microsoft Word.

### 2.4. Data Analysis

Initial data analysis aimed to identify any common patterns of responses among the pharmacists. The qualitative data were then assessed using content analysis according to the themes and subthemes of the interview questions so that responses could be compared between the CP neighbourhood leads. A summary of results was also provided in the form of a table, including frequency counts of responses to highlight the most common responses. This was completed by one member of the research team (MP), with another member (RM) reviewing all transcripts to ensure the accuracy of findings. Analysis was completed manually. Transcripts were read to ensure there were no transcription errors and then read again to enable immersion. Once interviews were finalised and transcribed, each participant was emailed a copy of their interview transcription to confirm they were happy with the recording.

The report includes direct quotations from the interview to explain the findings.

### 2.5. Ethics

This study received ethical approval (1213/045) from the delegated ethical approval team operating under the Kingston University Ethics Committee.

## 3. Results

All CP neighbourhood leads working in Lambeth participated in the study (n = 8). The sample population consisted of an equal number of male (n = 4) and female (n = 4) participants. The duration of time spent in the role as a CP neighbourhood lead was variable, with half the leads employed in the role for one to two years (n = 4)’ three participants in the role for over two years; and one participant in the role for only one year. The mean overall time spent in the role was 2.6 years. Many of the pharmacists held over 15 years of experience as a pharmacist (n = 5)

### 3.1. The Role of a CP Neighbourhood Lead

The participant responses explaining the role of a CP neighbourhood lead were variable. However, the main role defined by six out of eight participants was to act as a liaison between GPs and community pharmacies/pharmacists within their neighbourhoods. This is to facilitate communication between both parties by acting as a single point of contact who is responsible for collecting information from monthly meetings and then distributing this information back to all pharmacies in their neighbourhood, as well as communicating information collected from pharmacies in their neighbourhood to the GP surgery.

“*My role is to be a source of collective information to share with the other pharmacies [and] to provide support and advice*”.

Participant 3.

“*So my role is to make sure that we all deliver the services, liaise with the pharmacists and make sure we are all up to date*”.

Participant 5.

Participant 2 explained that the main aspect of acting as this channel between the leads and community pharmacies involves mediating.

“*My main role is to be a mediation between the GP surgery and community pharmacies*”.

Participant 2.

The role of mediation was echoed by other participants, as they reported needing to identify any issues experienced by pharmacies delivering commissioned services in the neighbourhood they are responsible for, identifying if there is anything that they can provide support with and feeding back these challenges to the CP neighbourhood leads, advocating on behalf of the pharmacies.

“*[My role is] communication, sharing information and data, providing support, understanding [pharmacists’] challenges, advocating on their behalf, thinking about what they what they face on a day-to-day basis and how to support them to address it, but at the same time, how to ensure that others are aware of the challenges they face*”.

Participant 6.

Four out of eight participants reported that their jobs as CP neighbourhood leads involved providing some form of support to the pharmacies and pharmacists in their neighbourhoods. This was mainly reported in the context of aiding with the delivery of commissioned services, for example, the CPCS, blood pressure checks and the flu vaccination service.

“*Mostly, at the moment, it’s to support [pharmacies] with the blood pressure service and the new services which are coming out. So, for example, previously we supported them to make the flu plan for the police service at our local PCN*”.

Participant 3.

Some participants (n = 3) noted that it is their role to ensure that pharmacies in their neighbourhoods are meeting the targets set out by the neighbourhood and ICB leads for commissioned services.

“*[My role is] basically just to meet the targets and make sure community pharmacies are reaching the targets*”.

Participant 4.

Participant 1 and Participant 3 noted that part of their job role is to promote local service delivery with general practices to offer local pharmacies more opportunities to provide commissioned services. A specific example given included promoting GPs’ trust in pharmacies so that they send more patient referrals to pharmacists through the CPCS.

“*So making sure the pharmacies action the referrals, trying to get the GPs to trust pharmacy so they send referrals. That’s part of what I have to do*”.

Participant 1.

“*Mostly at the moment, [my role is] to support [pharmacies] with the blood pressure service and the new services which coming out” “… and to help the local GP surgeries as well to help encourage services to be offered to other pharmacies*”.

Participant 3.

### 3.2. Number of Pharmacies and Pharmacists Connected in the Neighbourhood

The number of pharmacies each CP neighbourhood lead is responsible for varies between three pharmacies and eight pharmacies. Similarly, the number of pharmacists each CP neighbourhood lead is connected with and provides support to varied between 3 pharmacists and 11 pharmacists (Table 1).

### 3.3. Main Stakeholders

All the participants identified pharmacists among the main stakeholders of a CP neighbourhood lead (n = 8). Also, all participants identified GPs as stakeholders (n = 8). Seven participants identified the ICB as a primary stakeholder (Table 1).

### 3.4. Communication between CP Neighbourhood Leads 

The CP neighbourhood leads reported that the main vehicle for communication between the pharmacy leadership team within the borough takes place at the monthly meetings held either face-to-face or online with the ICB medicines team and other members of the neighbourhood, such as GPs, other CP neighbourhood leads and the LPC team. This meeting primarily focuses on providing the CP neighbourhood leads with a report on the performance status of each pharmacy within the neighbourhood they represent, specifically reporting on whether pharmacies are progressing in the delivery of commissioned services. The ICB medicines team provides data and updates, and the leads can understand relative performance through a benchmarking approach. The CP neighbourhood lead will then communicate this information to each pharmacy they are connected to locally in their neighbourhood. This allows the CP neighbourhood leads to identify which pharmacies require more support to fulfil service targets. Two CP neighbourhood leads reported that they use these meetings and data to identify which pharmacies in their PCN require support and focused attention.

“*So every month we have a meeting and the ICB leads present us the General Practice referrals summary and they look at the increases [and] they say which pharmacies are the lowest performing ones… The ICB reports pharmacies data back to us and ask us if we can have a word with them if they aren’t actioning referrals for example*”.

Participant 1.

One CP neighbourhood lead explained that these monthly meetings are an opportunity to collect information from other CP neighbourhood leads in the borough of Lambeth and use the information to compare the performance of his own neighbourhood against others and understand why and how another neighbourhood may be performing better or worse. Information shared between members of the neighbourhood and ICB provides guidance on how to improve the performance of the pharmacies they are responsible for.

“*So the monthly meetings, with the ICS… they’re effectively where we’re able to gather a lot of the information about where things are, allows us to be able to benchmark our PCN against other PCNs, understand some of the challenges that others are facing, understand where others have been more successful, and use that as part of the sharing exercise within the PCN itself. That information is then shared to your pharmacies*”.

Participant 6.

Two participants explained that they also discuss progress directly with the pharmacies within their neighbourhood and gain insight into the pharmacies’ own perceptions of their progress and how they are dealing with the demands of providing commissioned services.

“*[I] then just call up pharmacies and ask how they are doing*”.

Participant 8.

All participants stated that meetings with the other leads and the ICB occur monthly, either face-to-face or online. Regarding the frequency of communication with other pharmacies and pharmacists in the neighbourhood, some participants did not provide this information, while other responses included communicating on an ad hoc basis; weekly to monthly; or when receiving a call from a pharmacist in need “*every now and then*” (Participant 8). A summary of responses regarding communication can be found in Table 2.

The methods used to communicate that were reported by the CP neighbourhood leads included email, the WhatsApp messaging service, telephone calls and face-to-face meetings. Emails, WhatsApp instant messaging applications and telephone calls were reported to be used by six participants each, and face-to-face meetings are utilised as a mode of communication by five participants.

WhatsApp, face-to-face meetings and telephone calls were stated to be the most effective methods of communication, each by six participants. Participant 3 was among those who found face-to-face meetings were the most effective method.

“*I think having … those face-to-face conversations can be really motivational from somebody who knows how it feels, whereas if you’re doing things over the phone, I don’t think it is effective*”.

Participant 3.

Participant 3 explained that having in-person meetings with other pharmacists in the neighbourhood allows for the opportunity to build a rapport with her colleagues and shows that, as the CP neighbourhood lead, who also works simultaneously as a community pharmacist, she understands the challenges one faces in the job role and in trying meeting the demands of commissioned services.

Participant 5 voted for email correspondence as the most effective method of communication.

“*I think an email to me would be effective as I can action it in my own time accompanied with a Google doc form to sign to show that you have read it*”.

Participant 5.

Several participants (n = 5) noted that emails are the least effective method of communication because of barriers, such as the busy nature of working as a community pharmacist with time constraints, which makes it difficult to read and reply to emails; delays in response time from other pharmacists in the neighbourhood; and emails being easily missed because of the high volumes regularly received.

“*It’s time and the fact that the pharmacy profession is currently under a lot of pressure. People don’t have time to talk on the phone, they won’t have time to read or respond to emails*”.

Participant 4.

One participant stated that, although face-to-face meetings are good, they are not feasible because of busy schedules.

“*Face-to-face is good too but it is hard to find a common time where both of you are available*”.

Participant 4.

Some participants also felt that telephone calls were ineffective because of long waiting times in general practice reception queues or needing to call back multiple times, as community pharmacists are extremely busy.

“*With telephone calls, sometimes people are busy, so you have to call multiple times or arrange a time when you can speak to someone*”.

Participant 6.

The difficulties experienced regarding delayed response times in email correspondence were echoed by one participant in using the WhatsApp messenger service.

“*If I send a WhatsApp message out to my group, there’s no guarantee that anyone from WhatsApp is going to respond. They may read it, but they might not come back to me about it*”.

Participant 3.

Other reported barriers to effective communication within the neighbourhood were that leads have no form of direct communication with GPs.

“*A direct line to GPs would be nice as it can be very hard to get through to them. Phone calls or direct messaging without reception teams and having to wait or speak to intermediates takes a very long time so I do hate having time wasted*”.

Participant 1.

Another participant found difficulty in attending monthly meetings with other CP neighbourhood leads and the ICB. Despite the ICB funding the CP neighbourhood leads’ time, finding appropriate locum cover is difficult.

“*The funding is there, which is great, to allow us to get locum to cover time. But we have a slight barrier in that it is very hard to get locum cover for a few hours a day. So if I was to book a locum, if I were to ask my company to book a locum, it would have to be for the whole day as it is hard to book someone for a few hours*”.

Participant 3.

Two participants found that the times when leadership meetings are scheduled in the evening pose a barrier to engaging in communication with fellow CP neighbourhood leads and the ICB for different reasons.

“*The last training they did I couldn’t make it as I work in a late pharmacy and training was at 7 p.m. It’s very difficult to get locum for a few hours in the evening especially if they have to travel*”.

Participant 4.

“*Email link with an invitation to a meeting but evening meetings is very difficult for me as I have long hours and a family*”.

Participant 5.

### 3.5. Support Requirements

All eight participants voiced that further training in their roles as CP neighbourhood leads would provide benefits, such as training in communication, use of technology and IT services; leadership training; and training to understand the wider health system and the incentive of developing and encouraging commissioned services.

“*I think in my role as a [CP neighbourhood lead], I would say [support needs] would probably be a communication/leadership workshop or training. Workshop scenarios would be good. With the flu, it took me 2.5 months to get results back on flu jab numbers delivery. By the end of it, I got fed up and it shouldn’t be so hard to get a response*”.

Participant 5.

“*I suppose, a bit of training in terms of understanding things from a wider scale, in terms of developing and pushing services*”.

Participant 7.

“*Also someone like me needs training on IT as I have never had training. Someone needs to teach me*”.

Participant 8.

As previously mentioned, many participants (n = 4) reported that telephone calls, although noted as an effective method of communication, bring challenges in terms of communication. In light of this, one participant suggested that having a direct form of communication with GPs and community pharmacists in the neighbourhood would be beneficial.

“*I think technology in a way that you could connect the General Practice surgery and pharmacies in a better way would be good*”.

Participant 1.

Other suggestions to support the neighbourhood role included providing e-learning; recorded training sessions; and meetings to enable CP neighbourhood leads who could not attend to remain informed.

Three participants reported that the funding for their role is difficult to claim as it requires calculating time spent working as the CP neighbourhood lead, and this is itself a consumption of time; hence, these participants had not made claims for any work they had carried out, and as such, logging work should be made easier. One participant felt there is a lack of trust in their role among their superiors. Another participant suggested that funding an entire day of protected time for the job role would enable CP neighbourhood leads to dedicate sufficient time to it.

“*With the funding, they should provide a whole day funding so I can actually dedicate more time doing this role. Finding a locum would also be easier*”.

Participant 4.

“*Also with the funding, it is ridiculous as I can’t calculate the length of time I have spent on phone calls or trying to get through to people. I don’t have time to log all the activity and there seems to be a lack of trust. I am a professional and they should trust us. It’s good that there are checks in place but they need to trust us to do our jobs*”.

Participant 8.

## 4. Discussion

This study found active engagement from CP neighbourhood leads across Lambeth and found that a variety of leadership approaches are currently employed by the leads in undertaking their roles. The main leadership themes identified in this study were communication, relationships, training, time constraints, the collecting and sharing of data, performance monitoring, the use of digital solutions and service planning and delivery.

The main positive outcomes shared by the participants focussed on the communication they had with GPs, pharmacy colleagues and the ICB medicines team. Highlights included monthly meetings; access to and the sharing of data and performance; engagement with GPs; and having funded time to perform their roles. However, there were barriers and challenges that impacted their ability to undertake their roles: communication methods, time constraints, pressures from other work commitments and processes such as claiming payments and digital skills. These highlighted where improvements can be made in supporting the CP neighbourhood leads to be more effective in their roles.

This study found that the main role of a CP neighbourhood lead is acting as a conduit for communication between GPs and community pharmacy teams and that the preferred communication methods of most CP neighbourhood leads in Lambeth are face-to-face meetings, text messaging services and telephone calls. Participants noted that face-to-face meetings allow responses to be instantaneous, as opposed to emails, and are better for building rapport with other healthcare professionals. Most communication is transmitted through non-verbal cues without linguistic content [25], and face-to-face requests are more likely met with compliance than emails and other written, text-based platforms [26,27]. Steps should, therefore, be taken to enable and support leads and GPs to engage more frequently in face-to-face settings.

Although face-to-face meetings were deemed the most effective method of communication by the majority, the quarterly face-to-face meetings hosted by the ICB medicines team in the evenings can be challenging after a long working day. A literature review of 22 studies investigating views on continuing professional development (CPD) in pharmacy professionals in Great Britain found that lack of time is a main theme in reported barriers [28]. Previous studies have shown that those pharmacists who do not receive cover to attend learning events during the day prefer to prioritise their personal lives over learning events outside of pharmacy operating hours, similar to this study [29,30].

Protected time during existing working hours for training is desirable to deliver improvements in job satisfaction and wellbeing. This has been shown in studies on physicians whereby protected time for other research or personal use led to better overall job satisfaction, reduced burnout and improved wellbeing [31,32]. This is supported by a meta-analysis that found long working hours adversely affect the health and wellbeing of workers, including effects on cardiovascular health; depression and anxiety; work-related stress; sleep and fatigue; and health behaviours, for instance, reduced physical activity [33].

Previous research exploring interprofessional communication training found that benefits can include improving the confidence of pharmacists in initiating communication with other healthcare professionals, thereby enabling pharmacists to become more effective members of the healthcare team. Furthermore, training can improve self-perceived capability and confidence and enable pharmacists to proactively communicate with other clinicians [34].

Helping leads work in a more flexible manner will be important to their effectiveness in their roles. This should be supported through a development programme that ensures LPC, employers and ICB colleagues can provide the required support to CP neighbourhood leads in delivering their role in a flexible way to suit their own needs and ways of working.

It is important for CP neighbourhood leads to be able to communicate and influence effectively so they can effect change both with their networks of pharmacies and their local GPs. Using data to demonstrate the impact of activity was identified by a participant as being particularly helpful, so sharing data and building confidence to do so is important in effecting change.

Establishing a direct line to GPs or technology that connects GPs and pharmacies in a better way will improve communication efficiency. A similar suggestion was previously made regarding a universal system of communication between pharmacists and other primary healthcare providers to improve workflow processes and improve community-based care [35]. In addition, a qualitative study exploring GPs and pharmacists’ views on integrating pharmacists into general practice emphasised a need to have direct access to GPs [36]. Therefore, this shows the challenges faced by pharmacists in communicating with GPs and other primary healthcare providers. CP neighbourhood leads have an opportunity to establish better lines of communication through their roles.

This study further identified the need for digital skills and information technology (IT) training. There is a significant reliance upon IT in pharmacies [37], and since the COVID-19 pandemic, pharmaceutical care and education have seen a significant increase in the use of digital technology [38]. Previous research has shown that there is variable knowledge and skills regarding IT among community pharmacists, thus revealing a disparity in digital literacy [37]. This was further highlighted in a systematic review exploring factors influencing the adoption of IT services by healthcare professionals where lack of familiarity with the technology was a key barrier [39]. As such, the increased uptake of technology in community pharmacies and healthcare nationally necessitates further IT training to improve confidence in the job role, enable competency with the IT used and improve efficiency. Therefore, professional digital skill training should be recommended and provided to community pharmacy teams where needed.

The process for claiming payments was another process barrier identified. Restructuring the programme to provide CP neighbourhood leads with a regular payment associated with the provision of a regular report, rather than claiming each activity undertaken, will provide greater trust and more flexibility for leads in performing their roles. This can be supported through regular meetings and sharing information, activity and outcomes between CP neighbourhood leads and system partners.

Based on the findings of this study, areas for focus in any future development programme will include communication and influencing skills, building confidence, managing time and providing high-quality data. The programme should be co-designed with participants and provided in a blended approach to manage the constraints on time. Face-to-face meetings with peers and wider system leads are clearly important and of value, so time should be maximised for activities relevant to in-person meetings. Shorter, more frequent online webinars or calls where issues can be tackled and shared quickly and effectively would make it easier for participants to engage and interact.

The limitations of this study should also be taken into consideration. Firstly, the study was based on a small sample population within London that accounts for 8 out of approximately 1250 PCN networks in England, and thus, the findings may not be representative of community pharmacies in general and may also be specific to Lambeth. Furthermore, the communication CP neighbourhood leads receive in Lambeth may vary greatly compared with other boroughs within London and nationally. Further investigations and evaluations of the themes identified in this report should be undertaken across a larger area to better understand the impact of developing leadership programmes supporting community pharmacy practices.

## 5. Conclusions

Clinical leadership is an essential component in the transformation of health and care services, linked to increased uptake of clinical interventions and improvement in both the quality and consistency of care. This includes the community pharmacy setting. Investing time and resources in providing effective leadership is, therefore, key to delivering the transformational change required to maximise opportunities within the CPCF and make the best use of pharmacies and their unique abilities in addressing health inequalities and poor outcomes for those with the greatest risks to their health.

However, community pharmacies, like other healthcare providers, are facing unprecedented demands on their time. Delivering more clinical services through the community pharmacy sector requires adaptation to current operating models; greater investment in developing the wider pharmacy team; and better use of technology in the day-to-day running of pharmacies. Without change, community pharmacies will struggle to deliver on the ambition of the CPCF and contribute to the wider NHS primary care strategy to improve access and outcomes.

Our findings have demonstrated that establishing a CP neighbourhood lead programme is challenging, with many being new to the role. As the NHS goes through further reorganisation, it is imperative that health and care systems do more to support community pharmacy integration and invest in local leadership at the neighbourhood level. A leadership development programme should be provided to support local leads in acquiring leadership skills and building confidence in their roles. Specific recommendations should be adopted to

- Provide a leadership development programme and training to support leads in a way that makes effective use of their time;
- Provide effective support to system leads in setting high-level objectives and trust CP neighbourhood leads to deliver on them;
- Provide regular benchmarked performance data;
- Facilitate improved communication between pharmacies and general practices;
- Accelerate improved access to digital infrastructure to facilitate easier communication and the sharing of digital records across community pharmacies and general practices;
- Provide the necessary training to support pharmacies in maximising the use of digital solutions for patient care.

The results of this study will inform ongoing improvements to the programme as it expands across South East London beyond the pilot phase, and future evaluation should be undertaken with this larger population to address current limitations.

## Figures and Tables

**Table 1:** Overview of community pharmacy neighbourhoods in Lambeth.

			Main Stakeholders						
Participant	Number of Pharmacies in Neighbourhood	Number of Pharmacists in Neighbourhood	Pharmacists	GPs	ICB Medicine Team	LPC	Other Neighbourhood Leads	GP Teams	PCN Clinical Director
Participant 1	5	6	✓	✓	✓			✓	
Participant 2	5	5	✓	✓	✓		✓		
Participant 3	4	6	✓	✓	✓				
Participant 4	8	11	✓	✓	✓			✓	
Participant 5	3	3	✓	✓	✓				
Participant 6	8	11	✓	✓	✓				✓
Participant 7	4	5	✓	✓	✓	✓			
Participant 8	5	7	✓	✓					

**Table 2:** Summary of communication.

Participant	Information Communicated	Frequency of Communication	Current Methods of Communication	Most Effective/ Preferred Methods of Communication	Least Effective Methods of Communication	Barriers to Communication	Additional Support Needs
**Participant 1**	GP referral summary Blood pressure referrals	Monthly basis or ad hoc	Email	WhatsApp	Emails	Not speaking directly to a GP and having to wait for a receptionist	Direct messaging to GPs or pharmacists IT training
**Participant 2**	Information on new services and reports on how the pharmacies are doing	Monthly	Emails WhatsApp group Face-to-face meetings Telephone	Face-to-face with surgery direct messaging for community pharmacy	Phone calls	Time People not working collaboratively Lack of incentive	Collective targets and incentives Direct messaging to GPs Training in our roles
**Participant 3**	Our performance, locally CPCS hypertension service	Monthly	Face-to-face meetings WhatsApp group	Face-to-face meetings	WhatsApp	Very hard to obtain locum cover for a few hours a day	More communication training Management training
**Participant 4**	CPCS	Monthly “every second Thursday”	WhatsApp group Emails Face-to-face meetings Telephone	WhatsApp	Emails	Time	Record evening sessions E-learning IT training Make claiming funding easier
**Participant 5**	Updates on data	Monthly	WhatsApp group Telephone	email	N/A	Time Evening meetings difficult because of long hours and family	Record meetings or send a summary Make claiming funding easier Communication/leadership workshop or training
**Participant 6**	CPCS data Data on blood pressure checks Share best practices and barriers experienced by others	Monthly	face-to-face meetings Telephone calls Emails	Speaking to people directly, whether that’s on the phone or face-to-face.	Telephone	On the phone, you have to call multiple times or arrange a time	Independent prescribing Communication skills More sharing of best practices
**Participant 7**	CPCS data Feedback from surgeries and pharmacies on how they are getting on	Weekly to monthly	WhatsApp Emails Telephone calls	Telephone calls	Emails	Getting through to pharmacies via phone	Understanding things from a wider perspective in terms of developing and pushing services
**Participant 8**	Updates on progress Issues from pharmacies	Monthly	Face-to-face meetings Telephone calls WhatsApp groups Emails	Telephone calls	Emails	Timeliness of information Deadlines too close	IT training Make claiming funding easier

## Data Availability

The data presented in this study are available upon request from the corresponding author. The data are not publicly available because of the confidentiality issues of the participants.

## Appendix A

*Interview questions*

1. How many pharmacists and pharmacies are you connected with in your primary care network?
2. What is your role as a Community pharmacy neighbourhood lead in this network?
3. How has any previous experience you have affected your confidence in your role?
4. Who are the main stakeholders you deal with regularly?
5. What information is currently communicated within the neighbourhood, and how often?
6. From the information you receive and share, what would you say is essential, and what is nice to have?
7. When thinking about communication in the neighbourhood, what do you think makes communication effective, and what are the current barriers?
8. What support do you need to help you in your role?
9. Do you have any other comments that you would like to add about communication within the neighbourhood, and your role?

## Appendix B. COREQ Checklist

	**Item No.**	**Guide Guides/Description**	**On Page No.**
		Domain 1: Research team and reflexivity	
Interviewer/facilitator	1	Which author/s conducted the interview or focus group? RM	Methods—4
Credentials	2	What were the researchers’ credentials? MP—MPharm student RM—PhD, MPharm FR—MPharm, MSc	Title page
Occupation	3	What was their occupation at the time of the study? MP—MPharm student RM—Associate professor FR—Associate director	Methods—4 and title page
Gender	4	Was the researcher male or female? Male (MP, FR); Female (RM)	Methods—4
Experience and training	5	What experience or training did the researcher have? MP—student training RM—9 years of prior experience in qualitative research	Methods—4
		Relationship with participants	
Relationship established	6	Was a relationship established prior to the study commencement? No	Methods—4
Participant knowledge of the interviewer	7	What did the participants know about the researcher? e.g., personal goals, reasons for conducting the research Participants were made aware this was part of a research study and emailed an information sheet outlining the aims and objectives of the study	Methods—4
	**Item No.**	**Guide Guides/Description**	**On Page No.**
Interviewer characteristics	8	What characteristics were reported about the interviewer/facilitator? e.g., bias, assumptions, reasons and interests in the research topic MPharm student	Methods—4
		Domain 2: Study design	
		Theoretical framework	
Methodological orientation and theory	9	What methodological orientation was stated to underpin the study? e.g., grounded theory, discourse analysis, ethnography, phenomenology, content analysis Content analysis	Methods—5
		Participant selection	
Sampling	10	How were the participants selected? e.g., purposive, convenience, consecutive, snowball Purposive	Methods—4
Method of approach	11	How were the participants approached? e.g., face-to-face, telephone, mail, email Face-to-face and telephone	Methods—4
Sample size	12	How many participants were approached? Eight were approached; eight were interviewed	Methods—4 Results—5
Non-participation	13	How many people refused to participate or dropped out? Reasons? No dropouts	Results—5
		Setting	
Setting of data collection	14	Where was the data collected? e.g., home, clinic, workplace Via Microsoft Teams or telephone	Methods—4
Presence of non-participants	15	Was anyone else present besides the participants and researchers? No other individuals were present	Methods—4
Description of sample	16	What are the important characteristics of the sample? e.g., demographic data, dates Interviews were conducted between February and March 2023 8 CP neighbourhood lead pharmacists	Methods—4 Results—4
		Data collection	
Interview guide	17	Were questions, prompts, guides provided by the authors? Was it pilot-tested? Semi-structured interviews were used. Questions were provided by the authors. Face validation received.	Methods—4
Repeat interviews	18	Were repeat interviews carried out? If yes, how many? No	
Audio/visual recording	19	Did the research use audio or visual recording to collect the data? All interviews were audio recorded and transcribed	Methods—4
Field notes	20	Were field notes made during and/or/after the interview or focus group? No additional notes were made	Methods—4
Duration	21	What was the duration of the interviews or focus groups? They lasted between 12 and 30 min	Methods—4
Data saturation	22	Was data saturation discussed? All those who agreed to participate were included	Methods—4
Transcripts returned	23	Were transcripts returned to participants for comments and/pr correction? No	
		Domain 3: analysis and findings	
		Data analysis	
Number of data coders	24	How many data coders coded the data? Transcripts were read by two members of the research team (MP, RM)	Methods—5
Description of the coding tree	25	Did authors provide a description of the coding tree? Questions were used as codes	Methods—5
Derivation of themes	26	Were themes identified in advance or derived from the data? Inductive content analysis was used	Methods—5
	**Item No.**	**Guide Guides/Description**	**On Page No.**
Software	27	What software, if applicable, was used to manage the data? Data were analysed manually	Methods—5
Participant checking	28	Did participants provide feedback on the findings? No	
		Reporting	
Questions presented	29	Were participant quotations presented to illustrate the themes/findings? Was each quotation identified? e.g., participant number Comments were supported with direct quotes from participants who were anonymised by their country or professional representation	Methods—5 Results—6–13
Data and findings consistent	30	Was there consistency between the data presented and the findings? Yes	Results—6–13
Clarity of major themes Clarity of minor themes	31 32	Were major themes clearly presented in the findings? Yes Is there a description of diverse cases or discussion of minor themes? No	Results—6–13
